# Swept-Source OCT Mid-Peripheral Retinal Irregularity in Retinal Detachment and Posterior Vitreous Detachment Eyes

**DOI:** 10.3390/bioengineering10030377

**Published:** 2023-03-19

**Authors:** Stewart R. Lake, Murk J. Bottema, Tyra Lange, Keryn A. Williams, Karen J. Reynolds

**Affiliations:** 1 Flinders Institute for Health and Medical Research, GPO Box 2100, Adelaide 5001, Australia; keryn.williams@flinders.edu.au; 2 Medical Device Research Institute, College of Science and Engineering, Flinders University, GPO Box 2100, Adelaide 5001, Australia; murk.bottema@flinders.edu.au (M.J.B.); karen.reynolds@flinders.edu.au (K.J.R.)

**Keywords:** Fourier analysis, machine learning, posterior vitreous detachment, optical coherence tomography, retinal detachment

## Abstract

Irregularities in retinal shape have been shown to correlate with axial length, a major risk factor for retinal detachment. To further investigate this association, a comparison was performed of the swept-source optical coherence tomography (SS OCT) peripheral retinal shape of eyes that had either a posterior vitreous detachment (PVD) or vitrectomy for retinal detachment. The objective was to identify a biomarker that can be tested as a predictor for retinal detachment. Eyes with a PVD (N = 88), treated retinal detachment (N = 67), or retinal tear (N = 53) were recruited between July 2020 and January 2022 from hospital retinal clinics in South Australia. The mid-peripheral retina was imaged in four quadrants with SS OCT. The features explored were patient age, eye axial length, and retinal shape irregularity quantified in the frequency domain. A discriminant analysis classifier to identify retinal detachment eyes was trained with two-thirds and tested with one-third of the sample. Retinal detachment eyes had greater irregularity than PVD eyes. A classifier trained using shape features from the superior and temporal retina had a specificity of 84% and a sensitivity of 48%. Models incorporating axial length were less successful, suggesting peripheral retinal irregularity is a better biomarker for retinal detachment than axial length. Mid-peripheral retinal irregularity can identify eyes that have experienced a retinal detachment.

## 1. Introduction

More than 50% of retinal detachments present with the macula detached [1]. Combined with surgical complications [2], most people who experience a retinal detachment will suffer permanent vision loss even with successful intervention [3]. The prevention of retinal detachment with cryotherapy or laser retinopexy has been shown to be effective post-posterior vitreous detachment (PVD) in the presence of a retinal tear [4] and prior to PVD in eyes known to be at high risk, including type 1 Stickler syndrome and the fellow eyes of individuals with a giant retinal tear [5,6,7,8,9,10,11]. Unfortunately, for the majority, there is no clinical feature to identify those who would benefit from prophylactic treatment. Increased axial length or myopia is not specific enough to guide intervention. Lattice degeneration, the most significant peripheral retinal degeneration, has also been found insufficient to guide treatment [12,13]. There is then the need for a biomarker to increase the identification of eyes that would benefit from prophylactic treatment prior to vision loss.

Optical coherence tomography (OCT) is widely available in ophthalmic practices [14] The retinal shape or contour, defined as the path of the retinal pigment epithelium across the B-scan, has already been shown to be a useful feature in retinal disease management. Macular curvature is useful in the assessment of myopic maculopathy, including dome-shaped maculopathy, myopic traction maculopathy, and macular schisis [15,16,17,18,19]. Smaller within-scan features have also been shown to be useful in the assessment of macular degeneration and focal choroidal excavation in pachychoroid retinopathy [20,21,22,23]. While the generation of the rectangular B-scan image from the fan-shaped A-scan capture leads to some alteration in general retinal curvature (which can be corrected) [24] the smaller within-scan features are well preserved in the image in a manner similar to the way in which the patterns on a leather belt remain recognisable, whether it is held straight or flexed.

Retinal irregularity is the difference between the best-fit curve to the retinal contour and the retinal shape. This can be quantified in the frequency domain through Fourier transformation, which deconstructs the irregularity into shape features consisting of partitions (bins) in a spectrum of sine waves of varying frequencies. Retinal irregularity varies in a consistent manner across different regions of the posterior and mid-peripheral retina and increases with increasing axial length, the primary determinant of myopia [25]. Differences in irregularity measured with spectral domain OCT (SD OCT) have been found between eyes with a retinal detachment and those with PVD [26]. Over the last decade, swept-source OCT (SS OCT) has emerged as a new imaging modality with its own unique properties [27,28,29]. SD OCT scan rates of 65,000 A-scans per second can be exceeded by swept-source devices, which may achieve up to 200,000 A-scans captured per second [30,31]. Swept-source OCT uses a tuneable laser to replace the SD OCT super-luminescent diode as the sampling light source, with the returning light from the object captured by a dual-balanced photodetector [32]. Ophthalmic SS OCT use lasers with wavelengths in the infrared, typically centred around 1040–1060 nm, longer than the SD OCT light source, which has the disadvantage of reducing its minimum theoretical axial resolution, although the fast resampling combined with image processing can offset this to produce results similar to SD OCT [33]. The advantages of swept-source OCT are a faster image capture rate and lower sensitivity roll-off with increasing tissue penetration, which provides greater image quality across the depth of tissue [34,35]. This has enabled longer and wider retinal sampling in a single B-scan [36,37,38]. This paper explores the use of SS OCT mid-peripheral retinal images in differentiating retinal detachment from PVD eyes. The aim is to identify a biomarker that can be tested prospectively to identify eyes at risk of retinal detachment before PVD occurs. As an initial step, the objective here was to find such a biomarker in eyes that have experienced PVD or treatment for retinal detachment or a retinal tear.

## 2. Materials and Methods

### 2.1. Subjects

Participants were recruited from outpatient eye clinics at two general teaching hospitals (Flinders Medical Centre and the Royal Adelaide Hospital) in South Australia. Eyes were imaged from individuals who had experienced either a PVD, retinal tear, or retinal detachment, with the latter two groups including only those that were the result of PVD-related events and after all treatment was completed. All eyes were examined by a retinal specialist (S.R.L.), and PVD was diagnosed in the presence of typical symptoms and signs and confirmed with OCT. All imaging was performed between July 2020 and January 2022 at Flinders Medical Centre using a Zeiss swept-source Plex Elite OCT (Carl Zeiss Meditec, Dublin, CA, USA). The study was approved by the Southern Adelaide Local Health Network Human Research Ethics Committee and was performed in accordance with the tenets of the Declaration of Helsinki. Prior written informed consent was obtained from all participants.

### 2.2. Image Capture

**Figure 1:**
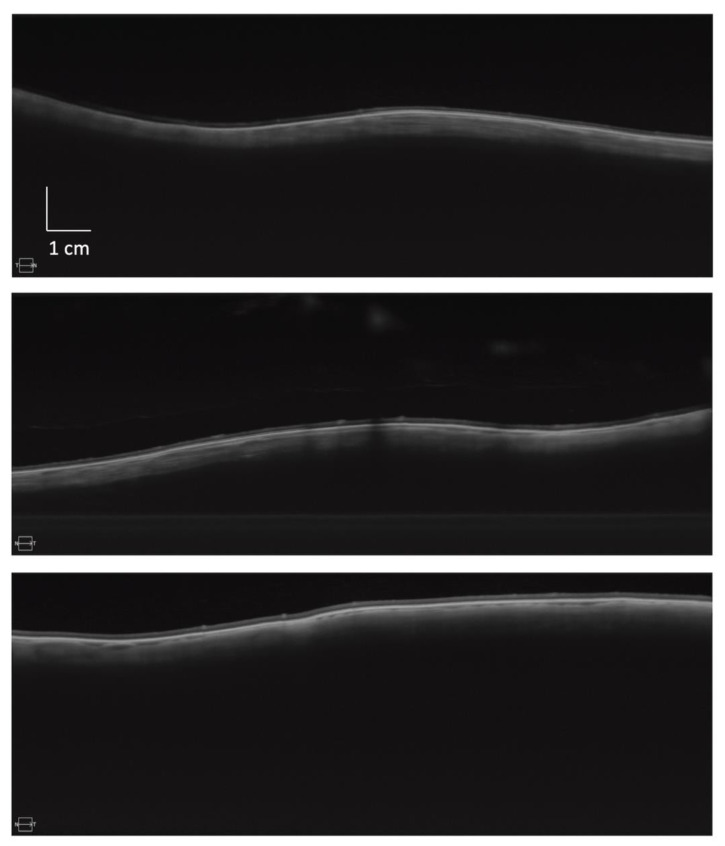
Sample swept-source OCT peripheral retinal images. Sample 16 × 6 mm^2^ B-scan mid-peripheral retinal images from three different eyes, illustrating variation in retinal contour. Each image is composed of 100 repetitions of the same scan.

SS OCT images were taken using a UHD 1 Spotlight 200 kHz scan, with a single 16 mm (2047 pixels) wide by 6 mm (3072 pixels) deep composite image created from 100 repetitions of the B-scan, eliminating the effects of subject movement. Four peripheral retinal images were taken from each eye. Images were taken in the coronal plane perpendicular to the direction of gaze, with the participant looking up, down, left, and right. The temporal and nasal scans were oriented vertically at 90 degrees to the horizontal, with those taken looking up and down parallel to the horizon. OCT image capture was performed at the most extreme eccentricity, where retina could still be visualised across the full width of the 16 mm B-scan window (Figure 1). The 6 mm deep scan ensured that all eyes were able to be imaged across the full width. 

### 2.3. Image Processing

Data extraction and image analysis were performed with programs written for this study in MATLAB. Raw .img data files were exported from the OCT device with the IMG export facility. These were converted to tagged image file format (tiff). Retinal shape represented by the retinal pigment epithelial line was extracted from the OCT images using a purpose-built graph theory algorithm [39]. The best-fit second-order polynomial curve was subtracted from the retinal contour, and a fast Fourier transformation was performed on the residual. The moduli for each frequency bin were corrected for the length of the signal (the adjusted length of the retina in the B-scan). This results in all irregularity values in the frequency domain being measured in mm^2^ per mm of retina imaged or simply mm. For each scan, the irregularity was determined relative to the average irregularity of the PVD eyes. PVD eyes alone were used for this average so that the reference standard came from the most common single diagnostic group. The eyes were distributed into five folds, and to ensure that there was an even distribution of axial lengths and diagnoses between each fold, they were sorted separately for each diagnostic group by axial length. Eyes were randomly allocated from each group into the 5 folds by generating consecutive sets of random numbers from 1–5 equal to the number of eyes. Numbers in each fold were non-equal when the sample was not divisible equally by five, and the remaining *n* samples were randomly placed into *n* groups. For eyes in each fold, an average B-scan irregularity was calculated from all the images from PVD eyes in the other 4 folds, equivalent to 80% of the PVD sample, and the difference between this and each image’s irregularity was determined. A candidate feature vector for each eye was created consisting of the first 30 bins of the irregularity spectrum from each of the four directions of gaze (in order: up, down, temporal, and nasal), the best-fit curvature to the retinal contour for each image in the same order, the axial length of the eye, and the participant’s age, resulting in 126 potential features [25].

### 2.4. Statistical Methods

Spearman’s rank correlation was used to assess the correlation between axial length and average irregularity, the irregularity for each region, and age, for all eyes combined and by diagnostic group. Between-group comparisons were performed using two-sample *t*-tests. Comparison of regional differences in irregularity was performed with one-way ANOVA. All analyses considered statistical significance to be reached when *p* < 0.05. Classifier sensitivity and specificity were calculated from the PVD and retinal detachment eye test set. Sensitivity was calculated as retinal detachment eyes labelled 2 (true positive) divided by total number of test set retinal detachment eyes. Specificity was the number of PVD eyes labelled 1 (true negative) divided by the total number of test set PVD eyes.

### 2.5. Feature Selection

Feature identification and training were performed with retinal detachment and PVD eyes. Retinal tear eyes were considered as eyes that would have developed a retinal detachment but for their timely presentation and were used as a second validation set. The retinal detachment and PVD eyes were randomly split 2/3:1/3 into training and testing sets, and feature selection was performed with the training set prior to testing. Multivariate feature selection with regularisation methods LASSO and elastic net were used to identify potential feature combinations [40]. Both LASSO and elastic net input variables were scaled to a mean of 0 with variance of 1 with elastic net α = 0.5 and ten-fold cross-validation to identify potential features.

Once a reduced feature set was obtained, all possible combinations of the remaining features were explored using quadratic discriminant analysis using the training set to identify classifiers with three or fewer features and the highest sensitivity for a specificity greater than 0.90. The high specificity threshold was selected to reduce the number of false positives (identifying a PVD eye as one with a retinal detachment), as labelling a PVD as retinal detachment was considered less acceptable than vice versa. Classifier performance was then evaluated with the test set.

## 3. Results

### 3.1. Subjects

Participant demographics and the number of eyes in each group are reported in Table 1. In total, 88 eyes with a PVD, 67 eyes with a retinal detachment (treated using vitrectomy without scleral buckling), and 53 eyes with a retinal tear were imaged. Age ranged from 47 to 84 years, with axial length from 22.14 to 27.27 mm. Imaging was uncomplicated in all eyes, with no significant media opacities noted in the PVD group and the vitreous cavity optically clear post-vitrectomy in retinal detachment eyes. Subjects with a retinal detachment were younger and had larger eyes than those who experienced PVD. Those who experienced a retinal tear had eyes with shorter axial lengths than those who presented with a retinal detachment.

### 3.2. Within-Eye Distribution of Irregularity

**Figure 2:**
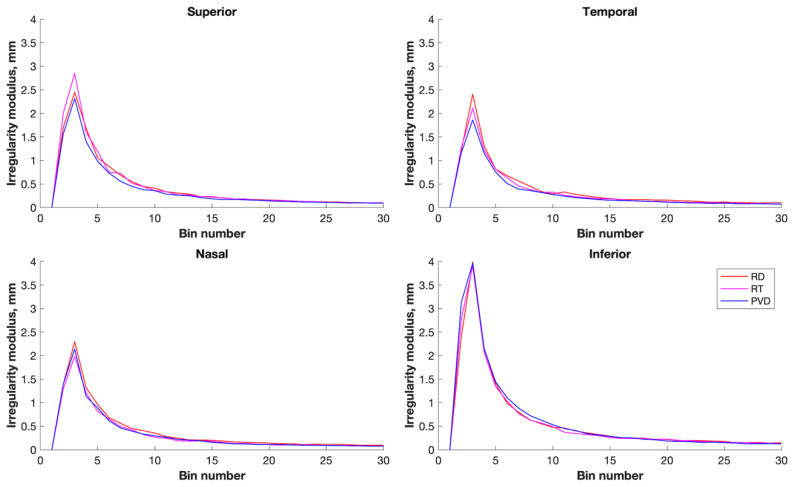
Regional irregularity, swept-source OCT. Average irregularity (presented here in frequency domain partitions (bins) for superior, temporal, inferior, and nasal retina) was greatest in the inferior retina for all diagnostic groups. PVD eyes (blue), retinal detachment eyes (red), and retinal tear eyes (pink). Irregularity units are mm^2^ per mm of retina, hence mm.

One-way ANOVA with a post hoc Tukey test indicated that the average irregularity was significantly greater in the inferior retina (mean (SD) 9.70 (7.14) mm) than in other regions (F (3,825) = 37.83, *p* < 0.001), with the temporal and nasal retinal irregularity (6.06 (2.51) mm and 6.01 (2.43) mm, respectively) no different from the superior retina (6.04 (3.16) mm) (Figure 2).

### 3.3. Between-Group Differences in Irregularity

Total irregularity did not differ between groups (retinal detachment irregularity 28.92 (8.14) mm, PVD 27.17 (8.72) mm, *p* = 0.21), with retinal tear irregularity intermediate between the two (27.49 (10.11) mm). Within each region, irregularity differed significantly between retinal detachment and PVD eyes in the superior retina (6.61 (3.44) mm vs. 5.53 (2.23) mm, *p* = 0.02) but not in any other retinal area.

### 3.4. Correlation of Irregularity with Axial Length

Axial length correlated weakly with the average total irregularity of all eyes (*p* = 0.02, *ρ* = 0.17) and average total irregularity for the PVD group alone (*p* = 0.04, *ρ* = 0.23) but not with average irregularity of the retinal detachment or retinal tear eye groups. Within the four individual regions, axial length correlated weakly with the total irregularity from superior (*p* = 0.05, *ρ* = 0.14) and inferior (*p* = 0.015, *ρ* = 0.18) retinas. In individual diagnostic groups, this correlation only persisted for PVD eyes (superior retina, *p* = 0.08, *ρ* = 0.20; inferior retina, *p* = 0.03, *ρ* = 0.24) and not with retinal detachment or retinal tear eyes.

### 3.5. Feature Selection

Elastic net regularisation identified a group of six shape features with a mean squared error = 0.22. These features were from lower frequency superior retina, axial length, lower frequency from the inferior retina, and three features from higher frequencies in the temporal retina. LASSO identified a group of three features with a mean squared error of 0.24. These three were also identified using elastic net, and all six features were further explored through the training set classifier performance.

### 3.6. Training Set Classifier Performance

All possible combinations of one–six features from the six candidate features were identified and used to train the quadratic discriminant classifiers. The three classifiers with the greatest training set sensitivity for specificity are shown in Supplementary Table S1: Performance of tested classifiers. The classifier generated from the fourth frequency bin from the superior retinal scan and two higher frequency bin (23 and 26) shape features from the temporal retinal scan were selected for use. Five-fold randomised cross-validation of the classifier using training set eyes repeated 20 times had an average success rate = 0.66, with the standard deviation of the success rates = 0.07.

### 3.7. Test Set Results

**Figure 3:**
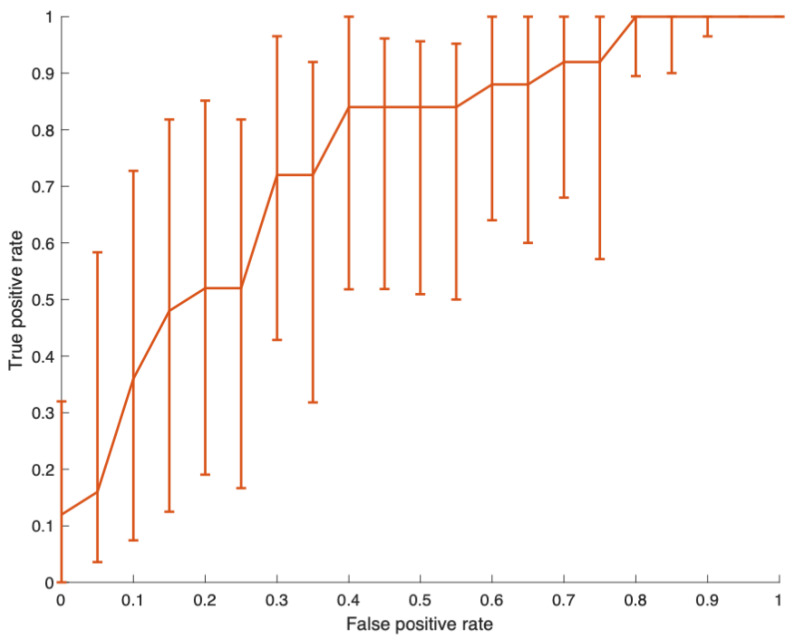
Classifier receiver operating characteristic curve. Vertical bars represent the 95% confidence intervals generated by 5000 bootstrap replicas. Classifier output centred to the median. Area under the curve = 0.74 (95% confidence intervals 0.59–0.85).

Table 2 presents the confusion matrix for the test set eyes. The classifier exhibited a specificity of 84% and a sensitivity of 48% in separating retinal detachment from PVD eyes. The initial receiver operating characteristic curve generated by 5000 bootstrap replicas showed an inverse sigmoid or logit shape, suggesting the predictor had a non-linear (U-shaped) relationship with the outcome [41]. This was corrected by centring the classifier output to its median value, leading to an area under the curve = 0.74 (95% confidence intervals 0.59–0.85, Figure 3). The classifier sensitivity for retinal tear eye identification was 35%.

## 4. Discussion

Mid-peripheral retinal shape irregularity identified eyes that had experienced a retinal detachment from a mixed sample of eyes that had experienced either PVD or PVD-related retinal detachment. Sensitivity for retinal detachment approached 50%, with a high specificity of 84%. An ability to identify half of the retinal detachment eyes is a considerable improvement on the status quo, where currently no test for retinal detachment is available. If this were to be employed as a test for retinal detachment, a high specificity is desirable to ensure eyes that are not at risk of vision loss are not mislabelled.

SS OCT retinal irregularity was greatest in the inferior retina and correlated with the axial length in eyes with a PVD. The lack of correlation between axial length and irregularity in retinal tear and detachment eyes, along with the slightly greater average irregularity, suggests that these eyes were simply more irregular regardless of size. The greater inferior irregularity was associated with a variety of retinal contours. In more than one-third of eyes, there was a localised infero-temporal concavity. These “micro-staphylomata” were up to 8 mm wide, but with a depth of less than 1 mm, they would be hard to identify using other imaging techniques.

The smallest changes that can be detected with SS OCT are defined by the spatial resolution of the images (an optical axial resolution of 6µm). However, this does not define changes corresponding to the beginning of retinal detachment. Classification is based upon the combination of three shape features in the frequency domain, which does not translate to a particular geometric change in the OCT. The threshold for each feature is not a single value and varies as the magnitude of the other two features changes.

### 4.1. Comparison with SD OCT

SS OCT retinal irregularity was greatest in the inferior retina, similar to previously reported SD OCT-determined irregularity [25]. Nasal and temporal retinal irregularities, here sampled vertically, were of a similar magnitude to superior retinal irregularity. This suggests that the lower magnitude irregularity seen in the temporal and nasal regions with SD OCT related more to the horizontal scan orientation rather than to regional differences between the superior and inferior retina and temporal and nasal retina and that mid-peripheral irregularity is greater when measured coronally compared with transversely.

The SS OCT classifier results are consistent with the reported findings with a classifier using SD OCT [26], with an increased sensitivity while maintaining high specificity. The improvement appears to be from the incorporation of shape features from coronal plane-imaged temporal retinas, a format that was not possible with the previous generation of SD OCT. Two of the six candidate features for classification (axial length and lower frequency upper retina) were consistent with the SD OCT classifier model. Perhaps surprisingly, in the context of the established association between myopia and retinal detachment, the model performed well without the inclusion of axial length, the only known useful anatomical metric for retinal detachment risk prior to retinal shape analysis.

The larger (16 mm) B-scan available with SS OCT enabled the analysis of shape features larger than was possible with SD OCT. These larger features (bins 2 and 3 in the frequency domain) were not important in classification, suggesting that the larger B-scan size is not critical when sampling shapes to classify retinal detachment and PVD eyes.

### 4.2. Association between Irregularity and Retinal Detachment

The cause of the association between axial length and retinal detachment and the link between this and local retinal shape are unknown. It is unlikely that irregularity leads directly to retinal tear formation but rather that an underlying property of the eye causes both increased shape irregularity and promotes retinal tear formation.

**Figure 4:**
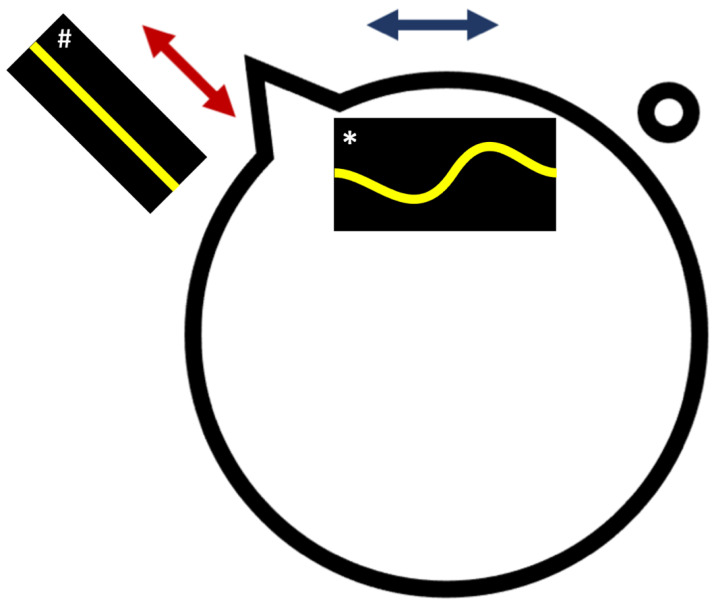
Hypothetical explanation for the association between retinal irregularity and retinal detachment. This schematic illustrates the posterior margin of the vitreous base (identified by the thick black line). Mid-peripheral retinal Bruch’s membrane grows in every direction during eye enlargement with myopisation. The limited growth of the eye in the coronal plane restricts Bruch’s membrane expansion, compressing it and leading to increased irregularity seen with retinal OCT (blue arrow and cartoon OCT with asterisk *). The greater growth of the eye in the antero-posterior direction leaves the retinal contour flattened or less irregular (cartoon OCT with hash mark #). The risk of retinal detachment arises from uneven antero-posterior Bruch’s membrane expansion drawing the posterior margin of the vitreous base posteriorly (red arrow), producing triangular protrusions or circular islets. These points are subject to increased mechanical stress during PVD, leading to retinal break or hole formation. The classifier identifies retinal detachment eyes by the coronal compression of Bruch’s membrane.

This link between local irregularity and retinal detachment may relate to regional variation in the growth of Bruch’s membrane in the equatorial regions of the eye [42,43,44]. Retinal breaks occurring with PVD are associated with a localised posterior extension of the posterior margin of the vitreous base (Figure 4) at or near the equator. Small segments of the posterior margin of the vitreous base may be drawn posteriorly with Bruch’s membrane expansion during myopisation, either in continuity with or separated from the continuous vitreous base. These posterior points of firm vitreo-retinal attachment are put under extra mechanical strain when PVD occurs up to the vitreous base, leading to hole or tear formation.

An expansion of Bruch’s membrane at the equator in the coronal plane may be limited by the more limited expansion of the eye size in this plane during myopisation [45]. Bruch’s membrane expansion may exceed the coronal circumference of the eye within which it is confined, leading to “wrinkles” in this plane, while axial expansion draws local segments of the vitreous base posteriorly, producing the configuration that leads to retinal breaks when PVD occurs (Figure 4). The relationship between retinal tear formation and irregularity is hypothesised to reflect an association arising from multidirectional but locally variable growth of the mid-peripheral Bruch’s membrane within an eye that grows axially more than coronally during myopisation.

Alternative explanations include alterations in connective tissue behaviour from collagen variations linked to myopia and retinal detachment-associated genes [46,47]. This may lead to both small-scale changes in scleral rigidity, producing shape irregularity, as well as abnormal vitreo-retinal attachment. Local variation in scleral strength may produce localised weakening and increased irregularity. These “micro-staphylomas” alter the interaction between the vitreous and retina, changing the strength of vitreo-retinal adhesion. In areas of the posterior extension of the vitreous base, this might lead to retinal tear formation when PVD occurs.

### 4.3. Limitations of This Study

As all groups analysed here are post-PVD, these results cannot establish that the same shape features prior to PVD will be able to predict retinal detachment. Prior work found no evidence of a change in retinal shape features with SD OCT compared with before and after PVD or retinal detachment surgery. Currently, fellow eyes without a PVD from individuals who have experienced a retinal detachment in one eye are being imaged to assess prospective model accuracy. The literature suggests the risk of retinal detachment in these eyes is 7–10% [48,49], with 42.4% of eyes that develop a PVD within five years of the cardinal event experiencing either a retinal tear or retinal detachment [50]. Classification accuracy may be improved further if clinical features, such as lattice degeneration, family history, and genetic profile, are considered [46,47,49,51,52]. Discriminant analysis is a suitable algorithm for a moderately-sized sample such as this, which may seem small for those used to deep learning models. All self-optimising classifiers are defined by their sample, so other populations may need their own training sets.

## 5. Conclusions

These SS OCT data provide further support for the concept that retinal shape differs between eyes that have had a retinal detachment and those that have experienced a PVD. The data presented here do not identify whether these shape features precede retinal detachment. However, SD OCT work has suggested that shape features are not affected by surgery, retinal detachment, or PVD [16]. Prospective data will be required to demonstrate whether these features identify eyes at risk of retinal detachment prior to PVD.

## Figures and Tables

**Table 1:** Participant demographics.

Group	N	OD/OS	M/F	Age ± SD (Years)	Axial Length ± SD (mm)
PVD	88	47/41	42/46	65.3 ± 6.1	24.41 ± 1.10
Retinal detachment	67	36/31	41/26	62.6 ± 8.5 *	25.10 ± 1.10 **
Retinal tear	53	30/23	37/16	64.1 ± 6.5	24.41 ± 1.12 ***

* Age*, p* = 0.04; ** axial length, *p* < 0.005, comparing PVD to retinal detachment. *** Axial length differed between retinal tear and retinal detachment eyes, *p* = 0.001, with no other difference between retinal tear eyes and the other groups (two-sample *t*-tests). SD, standard deviation; OD, right eye; OS, left eye; M/F, male and female sex, respectively.

**Table 2:** Confusion matrix for swept-source OCT classifier.

	Label 1	Label 2	Total
PVD	26	5	31
Retinal detachment	13	12	25
Total	39	17	56

Numbers represent eyes from the validation set. This classifier was the second line in additional file Table 1. Sensitivity = 0.48, specificity = 0.84, Fisher’s exact test, *p* = 0.018.

## Data Availability

The datasets used in this study are available from the corresponding authors upon reasonable request. The code used in this study is available at https://theses.flinders.edu.au/view/48efac34-4de9-4113-bc6d-930b7b8c5a0e/1. URL accessed on 10 February 2023.

## Supplementary Materials

The following supporting information can be downloaded at: https://www.mdpi.com/article/10.3390/bioengineering10030377/s1. Supplementary Table S1: Performance of tested classifiers.

## References

[1] Van de Put M.A.J., Hooymans J.M.M., Los L.I. (2013). The Incidence of Rhegmatogenous Retinal Detachment in The Netherlands. Ophthalmology.

[2] Xu Z.Y., Azuara-Blanco A., Kadonosono K., Murray T., Natarajan S., Sii S., Smiddy W., Steel D.H., Wolfensberger T.J., Lois N. (2021). New Classification for the Reporting of Complications in Retinal Detachment Surgical Trials. JAMA Ophthalmol..

[3] Du Y., Mo X.-H., Li X.-L., Zeng J., Luo W., Huang M.-L. (2019). Vision-related quality of life and depression in rhegmatogenous retinal detachment patients. Medicine.

[4] Straatsma B.R. (1980). Peripheral retinal tears: Classification, prevalence and principles of management. Aust. J. Opthalmol..

[5] Fincham G.S., Pasea L., Carroll C., McNinch A.M., Poulson A.V., Richards A.J., Scott J.D., Snead M.P. (2014). Prevention of retinal detachment in Stickler syndrome: The Cambridge prophylactic cryotherapy protocol. Ophthalmology.

[6] Verhoekx J.S.N., van Etten P.G., Wubbels R.J., van Meurs J.C., van Overdam K.A. (2020). Prophylactic laser treatment to decrease the incidence of retinal detachment in fellow eyes of idiopathic giant retinal tears. Retina.

[7] Wolfensberger T.J., Aylward G.W., Leaver P.K. (2003). Prophylactic 360° cryotherapy in fellow eyes of patients with spontaneous giant retinal tears. Ophthalmology.

[8] Ripandelli G., Rossi T., Cacciamani A., Scarinci F., Piaggi P., Stirpe M. (2016). Laser prophylactic treatment of the fellow eye in giant retinal tears: Long-Term Follow-up. Retina.

[9] Morris R.E., Kuhn F., Sipos T. (2022). Preventing Retinal Detachment: Where are We? Implications from Stickler Syndrome. Clin. Ophthalmol..

[10] Naravane A.V., Belin P.J., Pierce B., Quiram P.A. (2022). Risk and prevention of retinal detachments in patients with stickler syndrome. Ophthalmic Surg. Lasers Imaging Retin..

[11] Linton E., Jalil A., Sergouniotis P., Moussa G., Black G., Charles S., Ivanova T. (2023). Laser Prophylaxis in Stickler Syndrome: The Manchester Protocol. Retina.

[12] Byer N.E. (1992). Rethinking prophylactic therapy of retinal detachment. Advances in Vitreoretinal Surgery.

[13] Wilkinson C.P. (2014). Interventions for asymptomatic retinal breaks and lattice degeneration for preventing retinal detachment. Cochrane Database Syst. Rev..

[14] Fujimoto J.G., Drexler W., Schuman J.S., Hitzenberger C.K. (2009). Optical Coherence Tomography (OCT) in ophthalmology: Introduction. Opt. Express.

[15] Shinohara K., Shimada N., Moriyama M., Yoshida T., Jonas J.B., Yoshimura N., Ohno-Matsui K. (2017). Posterior Staphylomas in Pathologic Myopia Imaged by Widefield Optical Coherence Tomography. Investig. Ophthalmol. Vis. Sci..

[16] Caillaux V., Gaucher D., Gualino V., Massin P., Tadayoni R., Gaudric A. (2013). Morphologic characterization of dome-shaped macula in myopic eyes with serous macular detachment. Am. J. Ophthalmol..

[17] Ohno-Matsui K., Fang Y., Shinohara K., Takahashi H., Uramoto K., Yokoi T. (2019). Imaging of Pathologic Myopia. Asia-Pac. J. Ophthalmol..

[18] Gaucher D., Erginay A., Lecleire-Collet A., Haouchine B., Puech M., Cohen S.Y., Massin P., Gaudric A. (2008). Dome-shaped macula in eyes with myopic posterior staphyloma. Am. J. Ophthalmol..

[19] Frisina R., Baldi A., Cesana B.M., Semeraro F., Parolini B. (2016). Morphological and clinical characteristics of myopic posterior staphyloma in Caucasians. Graefes Arch. Clin. Exp. Ophthalmol..

[20] Naz S., Ahmed A., Akram M.U., Khan S.A. Automated segmentation of RPE layer for the detection of age macular degeneration using OCT images. Proceedings of the 2016 Sixth International Conference on Image Processing Theory, Tools and Applications (IPTA) 1–4 (IEEE, 2016).

[21] Srinivasan P.P., Kim L.A., Mettu P.S., Cousins S.W., Comer G.M., Izatt J.A., Farsiu S. (2014). Fully automated detection of diabetic macular edema and dry age-related macular degeneration from optical coherence tomography images. Biomed. Opt. Express.

[22] Kafieh R., Rabbani H., Abramoff M.D., Sonka M. (2013). Curvature correction of retinal OCTs using graph-based geometry detection. Phys. Med. Biol..

[23] Xu H., Zeng F., Shi D., Sun X., Chen X., Bai Y. (2014). Focal Choroidal Excavation Complicated by Choroidal Neovascularization. Ophthalmology.

[24] Kuo A.N., McNabb R.P., Chiu S.J., El-Dairi M.A., Farsiu S., Toth C.A., Izatt J.A. (2013). Correction of ocular shape in retinal optical coherence tomography and effect on current clinical measures. Am. J. Ophthalmol..

[25] Lake S., Bottema M., Williams K., Reynolds K. (2019). The correlation between optical coherence tomography retinal shape irregularity and axial length. PLoS ONE.

[26] Lake S.R., Bottema M.J., Williams K.A., Lange T., Reynolds K.J. (2022). Retinal Shape-Based Classification of Retinal Detachment and Posterior Vitreous Detachment Eyes. Ophthalmol. Ther..

[27] Meleppat R.K., Fortenbach C.R., Jian Y., Martinez E.S., Wagner K., Modjtahedi B.S., Motta M.J., Ramamurthy D.L., Schwab I.R., Zawadzki R.J. (2022). In Vivo Imaging of Retinal and Choroidal Morphology and Vascular Plexuses of Vertebrates Using Swept-Source Optical Coherence Tomography. Transl. Vis. Sci. Technol..

[28] Meleppat R.K., Zhang P., Ju M.J., Manna S.K.K., Jian Y., Pugh E.N., Zawadzki R.J. (2019). Directional optical coherence tomography reveals melanin concentration-dependent scattering properties of retinal pigment epithelium. J. Biomed. Opt..

[29] Kolokoltsev O., Gómez-Arista I., Treviño-Palacios C.G., Qureshi N., Mejia-Uriarte E.V. (2016). Swept Source OCT Beyond the Coherence Length Limit. IEEE J. Sel. Top. Quantum Electron..

[30] More S., Kubach S., Gregori G., Shen M., Wang L., Jiang X., Laiginhas R., Shi Y., De Sisternes L., Rosenfeld P.J. (2021). Comparison of retinal pigment epithelium elevation between scans acquired at 200 kHz and 100 kHz rates. Investig. Ophthalmol. Vis. Sci..

[31] Akman A., Akman A., Bayer A., Nouri-Mahdavi K. (2018). Optical Coherence Tomography: Manufacturers and Current Systems. Optical Coherence Tomography in Glaucoma.

[32] Adhi M., Liu J.J., Qavi A.H., Grulkowski I., Lu C.D., Mohler K.J., Ferrara D., Kraus M.F., Baumal C.R., Witkin A.J. (2014). Choroidal Analysis in Healthy Eyes Using Swept-Source Optical Coherence Tomography Compared to Spectral Domain Optical Coherence Tomography. Am. J. Ophthalmol..

[33] Zheng F., Zhang Q., Shi Y., Russell J.F., Motulsky E.H., Banta J.T., Chu Z., Zhou H., Patel N.A., de Sisternes L. (2019). Age-dependent Changes in the Macular Choriocapillaris of Normal Eyes Imaged With Swept-Source Optical Coherence Tomography Angiography. Am. J. Ophthalmol..

[34] Ruiz-Medrano J., Flores-Moreno I., Montero J.A., Duker J.S., Ruiz-Moreno J.M. (2015). Morphologic features of the choroidoscleral interface in a healthy population using swept-source optical coherence tomography. Am. J. Ophthalmol..

[35] Minami S., Ito Y., Ueno S., Kataoka K., Takeuchi J., Ito H., Nakano Y., Kitagawa M., Leahy C., Straub J. (2020). Analysis of macular curvature in normal eyes using swept-source optical coherence tomography. Jpn. J. Ophthalmol..

[36] Choma M.A., Sarunic M.V., Yang C., Izatt J.A. (2003). Sensitivity advantage of swept source and Fourier domain optical coherence tomography. Opt. Express.

[37] Klein T., Wieser W., Eigenwillig C.M., Biedermann B.R., Huber R. (2011). Megahertz OCT for ultrawide-field retinal imaging with a 1050nm Fourier domain mode-locked laser. Opt. Express.

[38] Kishi S. (2016). Impact of swept source optical coherence tomography on ophthalmology. Taiwan J. Ophthalmol..

[39] Lange T., Lake S., Reynolds K., Bottema M. Automated Computational Diagnosis of Peripheral Retinal Pathology in Optical Coherence Tomography (OCT) Scans using Graph Theory. Proceedings of the 2020 Digital Image Computing: Techniques and Applications (DICTA) 1–3 (2020).

[40] Zou H., Hastie T., Tibshirani R. (2007). On the “degrees of freedom” of the lasso. Ann. Stat..

[41] Ho K.M. (2017). Effect of non-linearity of a predictor on the shape and magnitude of its receiver-operating-characteristic curve in predicting a binary outcome. Sci. Rep..

[42] Jonas J.B., Ohno-Matsui K., Panda-Jonas S. (2019). Myopia: Anatomic Changes and Consequences for Its Etiology. Asia-Pac. J. Ophthalmol..

[43] Jonas J.B., Jonas R.A., Bikbov M.M., Wang Y.X., Panda-Jonas S. (2022). Myopia: Histology, clinical features, and potential implications for the etiology of axial elongation. Prog. Retin. Eye Res..

[44] Jonas J.B., Ohno-Matsui K., Jiang W.J., Panda-Jonas S. (2017). Bruch membrane and the mechanism of myopization: A new theory. Retina.

[45] Atchison D.A., Jones C.E., Schmid K.L., Pritchard N., Pope J.M., Strugnell W.E., Riley R.A. (2004). Eye Shape in Emmetropia and Myopia. Investig. Ophthalmol. Vis. Sci..

[46] Boutin T.S., Charteris D.G., Chandra A., Campbell S., Hayward C., Campbell A., Nandakumar P., Hinds D., UK Biobank Eye & Vision Consortium, 23andMe Research Team (2020). Insights into the genetic basis of retinal detachment. Hum. Mol. Genet..

[47] Johnston T., Chandra A., Hewitt A.W. (2016). Current Understanding of the Genetic Architecture of Rhegmatogenous Retinal Detachment. Ophthalmic Genet..

[48] Mitry D., Singh J., Yorston D., Siddiqui M.A.R., Murphy A.L., Wright A.F., Fleck B.W., Campbell H., Charteris D.G. (2012). The fellow eye in retinal detachment: Findings from the Scottish Retinal Detachment Study. Br. J. Ophthalmol..

[49] Törnquist R., Stenkula S., Törnquist P. (1987). Retinal detachment. Acta Ophthalmol..

[50] Wallsh J.O., Langevin S.T., Kumar A., Huz J., Falk N.S., Bhatnagar P. (2023). Fellow Eye Retinal Detachment Risk as Stratified by Hyaloid Status on Optical Coherence Tomography. Ophthalmology.

[51] Burton T.C. (1989). The influence of refractive error and lattice degeneration on the incidence of retinal detachment. Trans. Am. Ophthalmol. Soc..

[52] Meguro A., Ideta H., Ota M., Ito N., Ideta R., Yonemoto J., Takeuchi M., Uemoto R., Nishide T., Iijima Y. (2012). Common Variants in the COL4A4 Gene Confer Susceptibility to Lattice Degeneration of the Retina. PLoS ONE.

