# Supplementary material for: Swept-Source OCT Mid-Peripheral Retinal Irregularity in Retinal Detachment and Posterior Vitreous Detachment Eyes
Source: Bioengineering (Basel). 2023 Mar 19;10(3):377. doi: 10.3390/bioengineering10030377 (PMC10044997; doi:10.3390/bioengineering10030377)
Supplement: Supplementary file 1 [file bioengineering-10-00377-s001.zip › bioengineering-2242516-supplementary.pdf]

Supplementary Materials:

Swept-Source OCT Mid-Peripheral Retinal Irregularity in Retinal Detachment and Posterior Vitreous Detachment Eyes

Table S1. Performance of tested classifiers.

	Training set eyes		Test set eyes		
**Features**	**Specificity**	**Sensitivity**	**Specificity**	**Sensitivity (RD)**	**Sensitivity (RT)**
4,79,86	0.912	0.366	0.95	0.36	0.41
4,83,86	0.912	0.390	0.84	0.48	0.35
79,86,125	0.930	0.366	0.90	0.45	0.25

Training then testing set performance for three quadratic discriminant analysis classifiers. The indices in the first column identify features by their position in the candidate feature vector (see Image Processing in Methods). Feature 4 is from lower frequency upper retina, features 79, 83, and 86 are higher frequency irregularity from temporal retina, and feature 125 is axial length. The classifier in the second row is described further in Table 2 and Figure 3. For this model, cross validation of the mean success rate of the training set = 0.66, with standard deviation of the success rates = 0.07. RD = retinal detachment, RT = retinal tear eyes.
